# The sex chromosomes of the aging epigenome

**DOI:** 10.18632/aging.104033

**Published:** 2020-08-30

**Authors:** Qihua Tan

**Affiliations:** 1 Epidemiology and Biostatistics, Department of Public Health, Faculty of Health Science, University of Southern Denmark, Odense C, DK-5000, Denmark

**Keywords:** aging, sex chromosomes, epigenetics, DNA methylation, X-chromosome inactivation

Sex chromosomes are specialized chromosomes that determine the sex of individuals. They differ from an ordinary autosome in form, size, and behavior. Importantly, the difference in sex chromosomes between males and females could impact aging and longevity in multiple ways [1]. Unfortunately, the sex chromosomes are almost ignored in genomic analysis of aging phenotypes due to technical and biological considerations. For example, current epigenome-wide association studies (EWAS) using microarray or sequencing technologies routinely drop data on the sex chromosomes to avoid complexity in data handling and statistical modeling. This resulted in a very limited literature on epigenetic studies on aging concerning the sex chromosomes. However, recent strategic analyses of DNA methylation data on relatively large cohorts have revealed distinct but critical roles of the sex chromosomes during the aging process.

The shortest in size but not least in importance. As the shortest of all human chromosomes, the Y-chromosome is generally dropped in sex combined EWASs due to limited information and reduced size of male-only samples. By focusing on relatively large DNA methylation datasets on blood samples, Jesper et al. [2] reported significant age-dependent methylation patterns on the Y-chromosome validated across multiple cohorts of the elderly. Different from the autosome that displayed a predominant pattern of decreased methylation levels with aging in the older cohorts [3], the Y-chromosome showed an overwhelmingly age-dependent hyper-methylation with an accelerating rate of increase in the higher age groups. Most interestingly, nearly all CpG sites hyper-methylated with age are negatively correlated with risk of death. It is thus postulated that the observed age patterns and their correlation with mortality could possibly represent an active response to the aging process by the blood cells that contribute to maintain male survival. In addition to age-related methylation changes, loss of Y-chromosome with aging has been associated with increased risk of death [4] which further exemplifies its importance in male aging and survival.

Dissecting the complexity in 2 versus 1. The X-chromosomes have been regarded as a “forbidden city” in epigenetic analysis due to sex differences in the epigenetic regulation on X-chromosome genes that impact sexual characteristics. When it comes to X-chromosomes, it is inevitable to consider X-chromosome inactivation (XCI), an event for equalizing the dosage of X-linked genes between males and females through epigenetic regulation with DNA methylation as the key mechanism. Because of that, epigenetic analysis on X-chromosomes ignoring XCI can be rendered invalid. Through strategic analysis of microarray DNA methylation data, Li et al. [5] very recently revealed different methylation patterns on the X-chromosomes in females by comparing site-specific methylation levels between sexes on data normalized and analyzed on males and females separately. The study showed that CpG sites escaping XCI are persistently de-methylated with increasing age in females with similar pattern observed in aging males. As these CpGs are enriched in the promoter regions, their age patterns could indicate enhanced functionality of the linked genes during the aging process independent of sex. On the contrary, CpGs under XCI in females are de-methylated with age in the younger cohort but start to become hyper-methylated with increasing age in the older cohort. The phenomenon could imply epigenetic regulation of male characteristics in young males by CpGs under XCI in females. The involvement of CpG sites (under and escaping XCI) in aging and survival merits careful investigations.

Challenges and perspectives. Similar to the association analysis on autosomal chromosomes, the observed patterns of age-dependent DNA methylation changes on sex chromosomes do not represent a causal relationship but just a correlation [6]. The fact that the predominant CpG sites hyper-methylated with age on the Y-chromosome and also on the autosomal chromosomes [7] display negative correlation with risk of death could suggest that the age-dependent methylation changes in the genome of blood cells are the response to aging rather than the cause of aging. As DNA methylation is tissue specific, it is also important to verify if the results from whole blood apply to other tissues. Furthermore, current results are based on data collected using methylation microarrays with limited coverage especially on the Y-chromosome. With fast development and wide applications of next-generation sequencing, more and more methylation sequencing data with high coverage will be available which could enable verifications of array-based findings and hopefully provide clues for exploring the paradox of sex difference in survival.
